# Imagination Reduces False Memories for Everyday Action Sentences: Evidence From Pragmatic Inferences

**DOI:** 10.3389/fpsyg.2021.668899

**Published:** 2021-08-20

**Authors:** María J. Maraver, Ana Lapa, Leonel Garcia-Marques, Paula Carneiro, Ana Raposo

**Affiliations:** CICPSI, Faculdade de Psicologia, Universidade de Lisboa, Lisbon, Portugal

**Keywords:** false memories, pragmatic inferences, imagination, retrieval, memory

## Abstract

Human memory can be unreliable, and when reading a sentence with a pragmatic implication, such as “*the karate champion hit the cinder block*,” people often falsely remember that the karate champion “*broke*” the cinder block. Yet, research has shown that encoding instructions affect the false memories we form. On the one hand, instructing participants to imagine themselves manipulating the to-be-recalled items increase false memories (imagination inflation effect). But on the other hand, instructions to imagine have reduced false memories in the DRM paradigm (imagination facilitation effect). Here, we explored the effect of imaginal encoding with pragmatic inferences, a way to study false memories for information about everyday actions. Across two experiments, we manipulated imaginal encoding through the instructions given to participants and the after-item filler task (none vs. math operations). In Experiment 1, participants were either assigned to the encoding condition of imagine+no filler; pay attention+math; or memorize+math. In Experiment 2, the encoding instructions (imagine vs. memorize) and the filler task (none vs. math) were compared across four separate conditions. Results from the two experiments showed that imagination instructions lead to better memory, by showing a higher proportion of correct responses and better performance in a memory benefit index. Similarly, a significant reduction of false memories was observed across both experiments, even though a complementary Bayesian analysis only supported this conclusion for Experiment 1. The findings show that imaginal encoding improves memory, suggesting the engagement of a distinctiveness heuristic and source-monitoring process.

## Introduction

Human memory can be untrustworthy. When reading the sentence “*the karate champion hit the cinder block*” we might very often infer that the cinder block was broken, although this outcome was not explicitly stated in the sentence. The generation of inferences depends on constructive non-intentional processes that often lead to memory errors and distortions (Carpenter and Schacter, 2017). The use of sentences embedded with pragmatic implications, such as the previous example is thus a useful way to induce false memories for everyday actions and to study the reconstructive nature of memory. This article explores the consequences of different encoding instructions in memory retrieval for information on day-to-day actions using the pragmatic inference paradigm. Given the damaging consequences of inaccurate memories, it is of great interest to understand the mechanisms behind memory for pragmatic inferences.

In pragmatic inference sentences, an implication normally occurs when, from the information presented, the reader expects something that was not explicitly stated or not necessarily implied, changing the original meaning of the sentence. In the example above, the sentence pragmatically implies that the cinder block was broken, although this consequence was never made explicit. A strategy to test if a sentence implies a pragmatic inference is whether it can be joined by a “but not” conjunction and result in a consistent sentence. That is, “*the karate champion hit the cinder block, but did not break it*.” The effectiveness of pragmatic inferences in eliciting false memories is well documented, since it has been repeatedly demonstrated that participants tend to falsely recall pragmatic implications of sentences (Brewer, 1977; Chan and McDermott, 2006; Carneiro et al., 2020). Moreover, they represent a sensitive and robust measure for the study of false memories since they allow for the dissociation between the semantic and episodic memory levels. In other words, what is inferred and remembered from a character in a sentence depends, in part, on what the reader knows about the characteristics of the character (Barclay et al., 1984). In the example, the term “*champion*” implies qualities of capability and strength, which may lead the reader to infer that he/she was able to break the cinder block when, in fact, he/she might not have. Therefore, although the inference might be semantically consistent with what is presented in the sentence, it was not explicitly stated at the episodic level (Brewer, 1977; Barclay et al., 1984; McKoon and Ratcliff, 1992; Graesser et al., 1994; Raposo and Marques, 2013).

It has been demonstrated that later memory recall for pragmatic inference sentences is sensitive to manipulations in the encoding phase, such as, for example, the repetition of the materials during study (McDermott and Chan, 2006), or whether subjects encode sentences using or not a semantic strategy (Barclay et al., 1984). Thus, despite the robustness of false memories, experimental manipulations in the way items are processed during encoding can modulate retrieval in different false memory paradigms. For example, imagining that an event might have happened can increase confidence that it really happened. This *imagination inflation* effect has been demonstrated for autobiographical memories (Hyman and Pentland, 1996; Mazzoni and Memon, 2003), as well as self-performed actions or actions performed by others (Goff and Roediger, 1998; Thomas and Loftus, 2002; Lindner and Echterhoff, 2015; Calvillo et al., 2019). Winograd et al. (1998) observed a positive correlation between false recall in the DRM paradigm (Deese, 1959; Roediger and McDermott, 1995) and individual differences in mental imagery (measured by the Vividness of Visual Imagery Questionnaire), suggesting that the more vivid the mental images, the more likely to be falsely remembered. The authors explain this effect by difficulties in source monitoring between the words presented externally and the internally generated images (Winograd et al., 1998). In contrast to the imagination inflation effect, several reports have demonstrated an effect of *imagination facilitation*. That is, imaginal encoding can increase correct recall and reduce memory errors. Imaginal encoding typically improves memory (Paivio, 1991), and specifically for false memories, deliberately generating images reduces false memories compared to a control condition in the DRM paradigm (Foley et al., 2006; Robin, 2011; Oliver et al., 2016). Of note, in the DRM, false memories are elicited by relational processing (i.e., a list level or inter-item effect; Hege and Dodson, 2004; Huff et al., 2020). Generating images during the encoding of DRM items (associative word lists or objects) seem to improve item-specific processing, thus decreasing the chances of activating the critical item (non-presented lure) during encoding, and enhancing retrieval of studied items, both in recall and recognition tests (Robin, 2011; Robin and Mahé, 2015). Moreover, according to Foley et al. (2006), imaginary strategies at encoding allow participants to benefit from a richer context for successful monitoring, which results in improved veridical memories and reduced false memories. Still other studies have shown that imaginal encoding has no effect on false recall (Newstead and Newstead, 1998).

Perhaps, the different outcomes that have been reported for the effect of imaginal encoding on false memories – in particular the imagination inflation and the imagination facilitation effects – might be explained by differences between the paradigms where this encoding strategy was employed. As described above, the imagination inflation effect was found when the imaginal encoding strategy was employed for autobiographical events and actions, while the imagination facilitation effect was found when such strategy was employed for verbal materials, such as the associative wordlists used in the DRM paradigm. Considering these divergent patterns of results across different false memory paradigms, it is of great interest to compile evidence and explore the impact of imaginal encoding using pragmatic inference sentences. This paradigm involves verbal information describing everyday events and actions. Similarly to the DRM, it elicits false memories for non-presented material. Yet, while the DRM effects occur at the inter-item (list) level, pragmatic inference effects do not rely on relational processing, occurring at an intra-item level.

In the present study, we wanted to extend the existing knowledge on the effects of different encoding instructions on false memories using a paradigm that resembles the type of memory errors generated for everyday actions. Here, we investigated how an imagination strategy at encoding affected false memories for information about everyday actions produced by pragmatic inferences. For that purpose, sixty pragmatic inference sentences were used. These sentences were presented in Portuguese since pragmatic inferences have been shown to be culture and language specific (Carneiro et al., 2020). Responses were coded following the standard criteria proposed by Brewer (1977), as detailed in the methods’ section. Across two experiments, the effect of imagination was studied by manipulating the instructions given to participants at the encoding phase as well as the after-item filler task (none, to allow time for participants to imagine vs. solving math operations). By manipulating the instruction task, we contrasted our experimental condition (imagination) against two control conditions: a memory condition, akin to those commonly used in memory research and an instruction to simply pay attention, to serve as a baseline. After encoding, participants either engaged in a no filler task, allowing elaborative rehearsal and the engagement in deeper processing or, alternatively, they performed math operations, which constrained rehearsal and elaboration (Craik and Watkins, 1973). This manipulation of the filler task is important as imaginal encoding is associated with rehearsal and elaboration, and as such it may depend on the time available for such processes to take place. Indeed, similar manipulations of the filler task have been repeatedly used for the study of different levels of processing in memory tasks (Lewandowsky and Oberauer, 2015; Bartsch and Oberauer, 2021), and specifically in false recall paradigms (Rhodes and Anastasi, 2000). Experiment 1 was a laboratory-based experiment, where participants were randomly assigned to one out of three conditions: imagine+no filler; pay attention+math; or memorize+math. In Experiment 2, we manipulated the encoding instructions (imagine vs. memorize) and filler tasks (none vs. math) orthogonally. Participants were randomly assigned to one of four conditions: imagine+no filler; imagine+math; memorize+no filler; or memorize+math. Based on the revised literature, we expected imagination to significantly affect false memory performance compared to our control instructions to memorize or pay attention to the sentences. We had two contrasting possible hypotheses regarding the direction of this effect. On the one side, one could expect imagination encoding to increase the proportion of false memories compared to the control encodings, as previously found for the performance of actions (Goff and Roediger, 1998; Thomas and Loftus, 2002; Lindner and Echterhoff, 2015; Calvillo et al., 2019). This imagination inflation effect would reflect an increased proneness to source-monitoring errors, due to the difficulty in differentiating between externally presented information (i.e., the information presented in the sentence) and internally generated images (i.e., imagining the information that is pragmatically inferred). Alternatively, as shown in the DRM, an instruction to imagine could reduce false memories, by promoting item-specific processing and decreasing the probability of generating a pragmatic inference during encoding. Moreover, the instruction to imagine may lead to a more deliberate consideration of what was presented (i.e., why the sentence included “hit” instead of “broke”), which could facilitate memory and monitoring compared to the control conditions. One possible mechanism supporting this could be the use of a distinctiveness heuristic (Dodson and Schacter, 2002). That is, the generation of images at encoding could provide more distinctive diagnostic cues that would help making monitoring decisions at the moment of retrieval (Dodson and Schacter, 2002; Hege and Dodson, 2004). The use of the distinctive heuristic for the reduction of false memories has been repeatedly demonstrated in the DRM paradigm (Dodson and Schacter, 2002; Hege and Dodson, 2004; Foley et al., 2006; Oliver et al., 2016). However, it remains unexplored how different encoding contexts would affect pragmatic inferences, a paradigm in which false memories are not generated from relational processing and resembles closely the type of memory errors that occur in everyday actions. Therefore, with the present study, we aimed to extend the current understanding on the effects of different encoding conditions in memory errors and to clarify the role of imagination in increasing or reducing errors generated by pragmatic inferences.

## Experiment 1

### Materials and Methods

#### Sample

A total of 120 participants (87 females; *M*_age_=25.63±7.76) voluntarily agreed to participate in a laboratory experiment and were rewarded with 10€ gift vouchers for their time. For both experiments, the sample size was determined based on the number of participants used in previous studies with similar designs (Carneiro et al., 2017, 2020; Soro et al., 2017) and depending on the availability of resources. Participants were randomly assigned to one out of three conditions based on the encoding instructions: imagine, memorize, or pay attention. All participants were provided with information from the study and gave informed consent according with the Declaration of Helsinki (World Health Organisation, 2013). All experimental procedures were approved by the Ethics Committee of the Faculty of Psychology of the University of Lisbon.

#### Materials and Procedure

Sixty pragmatic implication sentences in Portuguese adapted by Carneiro et al. (2020) were used in the current experiment. Participants performed their task individually in a laboratory computer and the experiment was programmed and ran in the online platform Qualtrics (Qualtrics, Provo, UT). Participants were randomly distributed to the experimental conditions. Likewise, the order of the sentences at the encoding and retrieval phases was randomized anew for each participant. Participants first performed a practice block of the encoding phase with five sentences (without pragmatic implications). After, during the encoding phase, participants were presented with individual sentences in the computer screen for 4.5s and were instructed to read and either memorize, imagine, or pay attention to them (i.e., “*The karate champion hit the cinder block*”). After each sentence, participants in the imagine condition were presented with a blank screen for 5s, while participants in the pay attention or memorize conditions had to solve a simple math operation for 5s (i.e., 25−9=?). We included a blank screen after the imagine condition to ensure that participants had enough time to engage in mental imagery. This is important given the well-established individual differences in the ability to generate mental images (Marks, 1973; Cui et al., 2007; Pearson et al., 2011). In the middle of the encoding phase, immediately after presentation of the 30th sentence, participants could take a self-paced break, before continuing with the encoding of the remaining sentences. To allow for this break, sentences were counterbalanced in two blocks of 30 sentences each. Two sets of materials were created to counterbalance the sentences across these two blocks. Sentences were randomized within each block. After the encoding of all 60 sentences, participants performed a distractor task for 5min, where they were asked to count the differences between four pairs of images. Finally, participants in all conditions performed a cued-recall test where the 60 sentences were presented, in a random order, without its critical words and participants had to fill in the gaps (i.e., “*The karate champion ____ the cinder block*”). The experiment lasted, on average, 40min to be completed. After completion of this experiment, participants took part in two additional experiments (independent of the current study) for 20min, totalizing a 60min experimental session, after which they were thanked and rewarded for their time.

Participants’ responses were recoded following an adaptation of the standard criterion proposed by Brewer (1977). Answers that match the original sentence or synonyms that maintained the meaning of the original sentence were considered correct responses, answers that matched the expected pragmatic inferences or their synonyms were considered pragmatic inferences responses, and other alternative answers were considered intrusions. Responses left blank were considered as omissions. For example, for the sentence “*the karate champion hit the cinder block*,” responses were classified as either: (a) correct responses (i.e., “*hit*”); (b) pragmatic inferences (i.e., “*broke/destroyed/crashed*”); (c) intrusions (i.e., “*kicked/lifted/moved*”); and (d) omissions (no response). Supplementary Table 1 includes the complete list of the 60 sentences used as well as their coding criteria.

We calculated the proportion of recall for the four different response types (correct responses, pragmatic inferences, intrusions, and omissions) by adding the number of each response type and dividing them by the total number of sentences (60). Table 1 summarizes the proportion of recall for each encoding condition group and response type.

**Table 1:** Mean proportions and standard deviations of the cued-recall tests for correct responses, pragmatic implications, omissions, and intrusions for the two experiments as a function of the experimental conditions.

	*N*	Correct responses	Pragmatic inferences	Intrusions	Omissions
**Experiment 1**					
Imagine+no filler	40	0.41 ± 0.16	0.36 ± 0.09	0.02 ± 0.03	0.05 ± 0.08
Memorize+math	40	0.20 ± 0.11	0.49 ± 0.09	0.03 ± 0.02	0.06 ± 0.13
Pay attention+math	40	0.25 ± 0.12	0.46 ± 0.11	0.02 ± 0.02	0.09 ± 0.12
**Experiment 2**					
Imagine+no filler	27	0.46 ± 0.14	0.38 ± 0.12	0.09 ± 0.05	0.07 ± 0.14
Imagine+math	29	0.34 ± 0.15	0.44 ± 0.12	0.12 ± 0.05	0.09 ± 0.10
Memorize+no filler	28	0.32 ± 0.15	0.45 ± 0.13	0.14 ± 0.08	0.08 ± 0.12
Memorize+math	29	0.29 ± 0.13	0.48 ± 0.14	0.14 ± 0.09	0.08 ± 0.11

### Results

#### Response Type

Statistical analyses were performed using IBM SPSS Statistics software version 25 (IBM Corp., Armonk, NY, United States). Because the four dependent variables (i.e., correct responses, pragmatic inferences, intrusions, and omissions) have different statistical distributions, we ran separate one-way ANOVAs for each response type, with the proportion of recall as the dependent variable and the encoding condition as the independent factor (imagine vs. memorize vs. pay attention). Results were further analyzed within the Bayesian hypothesis testing framework to quantify the evidence for differences between the conditions (*H*_1_) in favor of no differences (*H*_0_). Bayesian ANOVAs and *t*-tests were run in JASP (JASP Team, 2020) using the default settings [*r* scale fixed effects=0.5, *r* scale random effects=1, samples=auto (10,000)]. In short, according to the Lee and Wagenmakers’ classification (Ly et al., 2016), Bayes factors (*BF*_10_) above 3 provide evidence in favor of *H*_1_, below 0.3 support *H*_0_, while intermediate values are interpreted as inconclusive (van Doorn et al., 2019).

Results for correct responses revealed a main effect of encoding condition, *F*(2,117)=28.89, *p*<0.01, $\eta_{p}^{2}$=0.33, *BF*_10_=1.41e+8. *Post-hoc* Bonferroni comparisons showed that the group instructed to imagine (*M*=0.41±0.16) recalled a higher proportion of correct responses than the groups instructed to memorize [*M*=0.20±0.11; *p*<0.01; 95% CI (0.14–0.28)] and to pay attention [*M*=0.25±0.12; *p*<0.01; 95% CI (0.09–0.23)]. No differences in correct responses were observed between the instruction to memorize and pay attention [*p*=0.26; 95% CI (−0.12–0.02)].

Regarding memory errors, results for pragmatic inferences responses revealed a main effect of encoding condition, *F*(2,117)=17.83, *p*<0.01, $\eta_{p}^{2}$=0.23, *BF*_10_=82080.45. *Post-hoc* Bonferroni comparisons showed that the group instructed to imagine (*M*=0.36±0.09) recalled a lower proportion of pragmatic inferences than the groups instructed to memorize [*M*=0.49±0.09; *p*<0.01; 95% CI (−0.18–−0.07)] and to pay attention [*M*=0.46±0.11; *p*<0.01; 95% CI (−0.15–−0.04)]. No difference in the recall of pragmatic implications was observed between the instruction to memorize and pay attention [*p*=0.55; 95% CI (−0.02–0.08)].

As for intrusions, results showed that the evidence for an effect of encoding condition was inconclusive, *F*(2, 117)=2.78, *p*=0.06, $\eta_{p}^{2}$=0.04, *BF*_10_=0.78; and results for omissions provided moderate evidence for no differences between the encoding conditions, *F*(2, 117)=1.18, *p*=0.31, $\eta_{p}^{2}$=0.20, *BF*_10_=0.21.

#### Memory Benefit Index

In addition to the analyses of the different response types, we derived a memory benefit index which captures the size of the difference between correct recall and recall of pragmatic implications for each sentence. We computed this index by subtracting the proportion of pragmatic inferences from the proportion of correct responses and this difference was divided by the overall recall (i.e., Correct responses−Pragmatic inferences) / (Correct responses+Pragmatic inferences). This index captures the effect of error production relative to correct recall, while controlling for overall memory performance. It is thus a more fine-graded measure of memory accuracy as it is independent of the total number of responses generated. A higher score means greater memory accuracy (with higher veridical recall and lower pragmatic inferences committed), and a lower score reflects lower memory accuracy (with lower correct recall and higher pragmatic inferences committed). This variable was introduced in a one-way ANOVA with encoding condition (imagine vs. memorize vs. pay attention) as the between-groups factor. Results revealed a main effect of condition, *F*(2,117)=29.34, *p*<0.01, $\eta_{p}^{2}$=0.33, *BF*_10_=2.72e+8, and *post-hoc* Bonferroni comparisons showed that the group instructed to imagine (*M*=0.04±0.32) outperformed the groups instructed to memorize [*M*=−0.43±0.24; *p*<0.01; 95% CI (0.32–0.63)] and to pay attention [*M*=−0.30±0.29; *p*<0.01; 95% CI (0.19–0.50)]. No differences in memory performance were observed between the instruction to memorize and pay attention [*p*=0.13; 95% CI (−2.82–0.25)].

### Discussion

Results show that the instruction to imagine benefitted memory performance compared to the instructions to memorize and pay attention. Participants showed a higher proportion of correct responses, and a lower proportion of pragmatic inference errors. We should note, however, that the group instructed to imagine had a longer time to rehearse because of the presentation of a blank screen after each sentence, while the memorize and pay attention groups had to perform math operations after each sentence and thus were exposed to a harder environment for rehearsal. To determine if the benefit of imagination comes from the longer time to rehearse or from a reliable effect of the instruction to imagine, we ran a second experiment in which we improved methodological control and compared four different conditions resulting from the crossing of two factors: the encoding instruction (memorize vs. imagine) and the filler task (none vs. math operations). For this second experiment, we decided to exclude the pay attention condition since it held no significant differences from the typical memory instruction.

## Experiment 2

### Materials and Methods

#### Sample

A total of 179 university students from the Faculty of Psychology of the University of Lisbon participated in the study and received course credits for compensation. The first 29 participants were tested in the experimental laboratory of the Faculty of Psychology. Due to COVID-19 outbreak and subsequent national lockdown, data collection moved to online for the remaining 150 participants. For online data collection and following previous work (Carneiro et al., 2020), several attention checks were introduced across the experiment (see procedure below) to guarantee that participants completed the task successfully and attentively. Sixteen participants failed one or more attention checks during cued recall, and their responses were excluded from further analysis. Moreover, other 50 responses had to be excluded for the following reasons: 40 participants indicated that they were not native speakers of European Portuguese, and 10 participants did not complete the experiment or were interrupted during the session. Therefore, the final sample consisted of 113 valid participants (85 online and 28 from the laboratory; 94 females; *M*_age_=20.65±6.37). To rule out possible differences in memory performance as a function of the testing setting, we ran independent samples *t*-test in the four dependent variables. Despite the unbalanced distributions of participants across settings (28 in the laboratory vs. 85 online), results provide evidence for no differences between participants tested in the laboratory and online: correct responses: *t*(111)=0.21, *p*=0.83, 95% CI (−0.06–0.07), *BF*_10_=0.23; pragmatic inferences: *t*(111)=−0.35, *p*=0.72, 95% CI (−0.07–0.05), *BF*_10_=0.24; intrusions: *t*(111)=0.25, *p*=0.80, 95% CI (−0.03–0.03), *BF*_10_=0.23; and omissions: *t*(111)=−0.03, *p*=0.97, 95% CI (−0.05–0.05), *BF*_10_=0.23.

Participants were randomly assigned to one out of four conditions resulting from the factorial combination of encoding instructions and filler tasks: imagine+no filler; imagine+math; memorize+no filler; or memorize+math. Similarly, all participants were provided with information from the study and gave written informed consent. All procedures were approved by the Ethics Committee of the Faculty of Psychology of the University of Lisbon.

#### Procedure

Materials and procedure were similar to those used in Experiment 1 with the following exceptions. First, online participants were instructed to perform the task in a quiet environment without interruptions. Second, several attention checks were introduced across the study to guarantee successful performance in the task (following Carneiro et al., 2020). At the encoding phase, participants either read instructions to imagine or to memorize the sentences and, after each sentence, were either presented with a blank screen for 5s or a math problem to solve, according to the condition. Two attention checks were presented at the encoding phase. These consisted in pressing an arrow button under 10s to resume the presentation of the sentences (a timer was displayed). To allow for these attention checks to appear amid encoding, the sentences were counterbalanced across three blocks of 20 sentences each: the first attention check appeared at the end of the first block, after the 20th sentence; the small break appeared amid the second block, after the 30th sentence; and the second attention check appeared before the final block, after the 40th sentence. Three sets of materials were created to counterbalance the sentences across the three blocks of the encoding phase, a first block of 20 sentences, a second block of 20 divided by a break after the 10th sentence, and a final block of 20 sentences. Within each block, sentences were presented in a randomized order. The distractor task and test phase were the same as those in the previous experiment, but three additional attention checks were added at test: these consisted of fragmented sentences, similar to those presented for recall, but explicitly asking participants to write a given word (seven, backpack, and red) in the response field. At the end of the experiment, participants were asked to rate their level of attention (*M*=5.80±0.75) and the quality of their data (*M*=5.15±1.16) in a 7-point Likert scale. The presentation order for the fragmented sentences at test was randomized. Experiment 2 took, on average, 40min to be completed.

### Results

An independent samples *t*-test revealed no difference in the overall math accuracy between the memorize (*M*=0.75±0.10) and imagine (*M*=0.74±0.09) encoding conditions, *t*(56)=0.25; *p*=0.76; 95% CI (−0.06–0.04), *BF*_10_=0.27.

Akin to Experiment 1, responses of the final cued-recall test were recoded and classified using an adaptation of the standard criterion proposed by Brewer (1977; see Supplementary Table 1).

#### Response Type

We conducted four separate two-way ANOVAs with 2 (encoding condition: imagine vs. memorize)×2 (filler task: no filler vs. math), one for each dependent variable. Performance on the different variables as a function of condition is included in Table 1.

Results for correct responses revealed a main effect of encoding condition, *F*(1,109)=12.56, *p*<0.01, $\eta_{p}^{2}$=0.10, *BF*_10_=41.78, suggesting that the groups instructed to imagine (*M*=0.40±0.16) recalled a higher proportion of correct responses than the groups instructed to memorize [*M*=0.30±0.14; *p*<0.01; 95% CI (0.04–0.15)]. There was also a main effect of filler task, *F*(1,109)=7.65, *p*<0.01, $\eta_{p}^{2}$=0.06, *BF*_10_=6.59, with no filler (*M*=0.39±0.16) leading to a better performance than doing math operations [*M*=0.31±0.14; *p*=0.01; 95% CI (0.01–0.13)]. Results showed moderate evidence for an interaction between the factors, *F*(1,109)=2.92, *p*=0.09, $\eta_{p}^{2}$=0.03, *BF*_10_=3.31. This means that while for participants solving math operations there is no conclusive evidence for a difference in the proportion of correct responses between the instruction to imagine (*M*=0.34±0.15) and to memorize (*M*=0.29±0.13), *t*(56)=1.32, *p*=0.19, 95% CI (−0.17–0.86), *BF*_10_=0.55; for participants that saw a blank screen after each sentence, those in the imagine condition had a higher proportion of correct responses (*M*=0.46±0.14), than instructed to memorize (*M*=0.32±0.15), *t*(56)=3.65, *p*<0.01, 95% CI (0.42–1.54), *BF*_10_=47.89.

For pragmatic inferences responses, results revealed a significant main effect of encoding condition, *F*(1,109)=5.20, *p*=0.02, $\eta_{p}^{2}$=0.05, *BF*_10_=1.48, suggesting that the group instructed to imagine (*M*=0.41±0.12) committed a significantly lower proportion of pragmatic inference errors than the groups instructed to memorize [*M*=0.47±0.17; *p*=0.03; 95% CI (−0.10–−0.00)]. A main effect of filler task was also observed, *F*(1, 109)=4.48, *p*=0.04, $\eta_{p}^{2}$=0.04, *BF*_10_=1.13; with the no filler condition (*M*=0.41±0.13) leading to lower levels of pragmatic inference errors than doing math operations [*M*=0.46±0.13; *p*=0.04; 95% CI (0.00–0.10)]. The interaction between encoding condition and filler task did not reach significance, *F*(1,109)=0.50, *p*=0.48, $\eta_{p}^{2}$<0.01, *BF*_10_=0.51. Yet, in Bayesian analyses, the evidence for these effects is deemed inconclusive, meaning that, with the current sample size, we did not have enough power to provide evidence for reliable effects (Ly et al., 2016; Quintana and Williams, 2018).

Lastly, results for intrusions revealed a main effect of encoding condition, *F*(1, 109)=10.31, *p*<0.01, $\eta_{p}^{2}$=0.08, *BF*_10_=12.17, revealing that the group instructed to imagine (*M*=0.10±0.05) committed a lower proportion of intrusion errors than the group instructed to memorize [*M*=0.14±0.08; *p*<0.01; 95% CI (−0.06–−0.02)]. No other significant results were found (all *p*s>0.29, *BFs*_10_<0.37). No significant differences were found for omissions (all *p*s>0.47, *BFs*_10_<0.15).

#### Memory Benefit Index

Similar to Experiment 1, we computed a memory benefit index that was analyzed in a two-way ANOVA, with 2 (encoding instruction: imagine vs. memorize)×(filler task: no filler vs. math) as between group factors. Consistent with Experiment 1, results revealed a main effect of encoding instruction, *F*(1,109)=9.77, *p*<0.01, $\eta_{p}^{2}$=0.08, *BF*_10_=12.04, meaning that participants instructed to imagine (*M*=−0.03±0.33) outperformed those instructed to memorize [*M*=−0.21±0.33; *p*<0.01; 95% CI (0.07–0.31)]. Results also revealed a main effect of filler task, *F*(1,109)=7.08, *p*=0.01, $\eta_{p}^{2}$=0.06, *BF*_10_=4.45, such that performing math operations after encoding each of the sentences led to worse memory performance (*M*=−0.20±0.34) than to stare at a blank screen without doing any explicit task for 5s [*M*=−0.04±0.32; *p*<0.01; 95% CI (−0.28–−0.04)]. Finally, the interaction between the factors did not reach the level of significance, with the Bayesian analysis revealing inconclusive evidence for the absence or presence of an interaction, *F*(1,109)=2.18, *p*=0.14, $\eta_{p}^{2}$=0.02, *BF*_10_=1.90.

### Discussion

Experiment 2 aimed at disentangling whether the benefits that imagination had on memory in Experiment 1 were solely due to this encoding strategy or if these benefits were promoted by the task participants carried out after the presentation of each sentence (no filler task, allowing for rehearsal, vs. math operations). To do so, in Experiment 2, both filler tasks were manipulated across different encoding strategies. Results were partly consistent with Experiment 1: imaginal encoding seems to be beneficial for memory, compared to a memory encoding strategy. The instruction to imagine promoted an overall better performance, with higher levels of correct responses and lower levels of intrusion errors, compared to an instruction to memorize.

## General Discussion

The purpose of this research was to explore the effect of imagination as an encoding strategy on false memories induced by pragmatic inference sentences. In Experiment 1, we observed that instructions to imagine improved overall memory performance, by enhancing the level of correct responses recalled and by reducing the proportion of pragmatic inference errors committed, compared to other encoding strategies. However, given the methodological confound derived from the longer time to rehearse in the imagination group against the other conditions, caution is warranted in the interpretation of the results. Experiment 2 addressed this methodological limitation and revealed an effect of imagination in increasing the proportion of correct responses and improving overall performance (memory benefit index), compared to the memorize condition. In addition, a significant reduction in pragmatic inference errors was also observed in the imagination condition, indicating a decrease in false memories. Yet, while results from the Bayesian framework provided support for the reduction of pragmatic inference errors in Experiment 1, results from Experiment 2 suggested that with the current sample size, we did not have enough power to claim for such an effect in the imagine, compared to the memorize condition.

Regarding the differences as a function of the filler task, we observed that participants in the imagine condition had a higher proportion of correct recall compared to the memory condition, when they saw a blank screen after each sentence. We must note that the blank screen displayed after each sentence had a fixed presentation time of 5s. Therefore, although participants had the same time to rehearse the sentence, it was in the imagine condition where rehearsal benefitted subsequent memory recall. Furthermore, the overall performance in solving math equations did not differ between the instruction to imagine or to memorize, suggesting that the amount of time and elaboration was the same for both encoding conditions, and it was the combination of the instruction to imagine and the time to rehearse that led to more accurate recall (Bower, 1970).

Imagination has long been recognized for its enhancing effects on veridical memory (Paivio, 1991), and imaginal encoding strategies are often used as a mnemonic aid that can lead to increases in the amount of information that can be stored and retrieved (Paivio, 1991). The effectiveness of instructions that invite participants to use imaginal encoding strategies in verbal learning has generally provided consistent, reliable, and substantial improvements in tests related to retention, recall performance, and recognition (Richardson, 1998). According to the DRM literature, imagination is also beneficial by reducing false memories: When participants are instructed to imagine objects that correspond to the presented word-list items or to images presented on the screen, veridical memory is improved, and false memories are reduced compared to control instructions (Hege and Dodson, 2004; Foley et al., 2006, 2009; Oliver et al., 2016). Our results replicated this imagination facilitation effect and extended it to pragmatical implications on everyday actions: Imaginal encoding improved veridical memory of the stated information.

Several explanations can be proposed for this result. On the one hand, according to the impoverished relational-encoding view, imaginal encoding improves item-specific processing and reduces relational processing of information and thus false memories (Hege and Dodson, 2004; Foley et al., 2006; Oliver et al., 2016). According to Hege and Dodson (2004), encoding images interfere with the encoding of relational information – the main cause of false memories in the DRM – so that critical lures are less likely to be falsely recalled at the final memory test, resulting in an improved memory performance. In our experimental design, it was not possible to directly measure what people were actually imagining, in order to assess if the generated images matched what was explicitly stated in the sentence. Yet, the observed imagination facilitation effect suggests that indeed, instructing participants to imagine the sentences promote item-specific processing and attenuate the probability of generating pragmatic inferences. It would be interesting for future studies to ask participants what they imagined and evaluate whether relational information had been encoded or not.

On the other hand, imagination instructions seem to promote encoding of the presented material, by providing more specific characteristics or diagnostic cues (Foley et al., 2009; Robin, 2011). These cues can later be used to make better source-monitoring decisions, that is, to accept (veridical) information about which one has more mental images and to disregard (wrong) information that lacks such cues. This process may rely on the distinctiveness heuristic (Hege and Dodson, 2004; McCabe and Smith, 2006; Oliver et al., 2016), by which source-monitoring decisions would be guided by distinctiveness. Because the non-imagined material lacks distinctive details, it would, therefore, be rejected (Foley et al., 2009). This interpretation is supported by the results of Rajaram’s study showing that the recognition of pictures compared to words is based on the retrieval of distinctive features from memory rather than the familiarity of the events (Rajaram, 1993). Yet, results from Robin (2011) showed that, even though imaginal encoding reduces false memories in free recall tests, it does not reduce false memories in recognition tests, suggesting that distinctiveness was not at play. According to Robin (2011), the benefits of imaginal instructions for memory could either stem from an enhancement of the specific characteristic of the information encoded that will act as cues, facilitating veridical retrieval and only by that avoiding errors or from the use of the distinctiveness heuristic only when participants can benefit from appropriate contextual support – as it is the case of cued-recall tests but not for recognition tests where participants are exposed to false information.

Besides this, the distinctiveness heuristic is an inferential strategy that comes into play during retrieval, when an individual fails to remember sufficient information about a past event (Dodson and Schacter, 2002; Hege and Dodson, 2004). The distinctiveness heuristic depends on individual source-monitoring processes and on metamemorial beliefs of what should be remembered (Johnson et al., 1993; Dodson and Schacter, 2002). In this regard, failing to remember inferable information about an event (expected, but not necessarily veridical) can sign that the event never occurred. Dodson and Schacter (2002) demonstrated that encoding images biased toward the use of a distinctiveness heuristic and, in fact, previous research as shown that the use of this heuristic reduces false memories both in recognition and in recall tests (McCabe and Smith, 2006; Robin, 2011). In our case, we believe that the experience of imagination provides at encoding rich information details, regarding both the stimuli and the residual traces of the mental operations performed at encoding that can be used, promoting item-specific processing with distinctive and diagnostic details. This leads to richer and more detailed representations in memory for the imagined action and, at retrieval, the use of the distinctiveness heuristic – as a metamemorial decision-based strategy – allows the exclusion of items that are recalled in the absence of such details. Therefore, imaginal encoding could lead to a more careful dissociation between explicitly stated vs. inferable information.

The Fuzzy Trace Theory (FTT) provides an alternative but complementary explanation to our findings since it distinguishes between the parallel encoding of two different types of memory traces. On the one hand, gist traces encode general meaning-based representations, and on the other, verbatim traces encode superficial representations based on perception. According to the FTT, memory performance is based on the retrieval of both verbatim and gist traces. While both types of traces can support the accurate memory reconstruction, gist-irrelevant information can co-occur with the activation of gist traces, but they are suppressed by the retrieval of verbatim traces (Reyna and Kiernan, 1994; Brainerd and Reyna, 2002). Thus, the memory errors generated by pragmatic inference sentences would be consistent with the retrieval of gist traces, since they represent a deductive interpretation of concepts (meanings, relations, and patterns) that integrate world knowledge with textual information (Kintsch, 1974; Reyna and Kiernan, 1994). In this sense, in our study, the imaginary activity generated at encoding promoted the processing of verbatim traces (i.e., episodically instantiated representations of the presented items), which resulted in the imagination facilitation effect, leading to an increase in correct responses and a reduction in pragmatic inference errors.

The imagination facilitation effect is very robust for DRM-induced false memories and, supported by our results, for pragmatic inference sentences. Nonetheless, there are reports of imagination inflation effects with other memory paradigms (Hyman and Pentland, 1996; Goff and Roediger, 1998; Mazzoni and Memon, 2003; Lindner and Echterhoff, 2015; Calvillo et al., 2019). Such discrepant results may be explained by differences in the paradigms employed. As mentioned at the outset, while the imagination facilitation effect was found using the DRM paradigm, that relies on verbal material and their semantic association, the imagination inflation effect was found for autobiographical events and actions. That is one of the reasons that makes the use of pragmatic inference sentences particularly interesting: These materials describe everyday actions and events but rely on semantic extraction and association; and yet the imagination facilitation effect persisted.

Another possible approach to the discrepant result patterns, less parsimonious but perhaps more conciliatory, is that these findings may reflect two sides of the same coin. As it was already stated, imagination leads to a better retrieval of the studied information (Paivio, 1991; Richardson, 1998; Foley et al., 2006). Perhaps imagination increases overall the retrieval of the imagined information – whether true (explicitly studied) or false (not presented). In the DRM paradigm and in the pragmatic inference paradigm employed here, participants are asked to imagine the presented words and sentences, respectively. So, presumably, they imagined veridical information, which in turn promoted veridical retrieval and reduced false memories for non-imagined lures or pragmatic implications. However, in the cases where an imagination inflation effect was found, participants were asked to imagine events and actions that they did not perform and were thus considered as false (not presented) information. Perhaps both inflation and facilitation effects reflect an increase of retrieval for all imagined information, true or false, as long as imagined. Our results provide evidence for a beneficial effect of imagination in memory using pragmatic inferences. However, caution should be warranted when generalizing our results to other materials and paradigms. Although we consider that in our data no additional analysis could allow us to differentiate between imagination inflation and facilitation, it might be interesting for future studies to compare memory performance between different types of pragmatic inference sentences, for example, manipulating the agent (i.e., third vs. first person), as this has been argued to be a critical factor in false memory paradigms on action performance (Lindner and Echterhoff, 2015). This would allow testing the extent to which our results (a facilitation effect) generalize to conditions similar to those used in other paradigms.

Some limitations of the current study are worth acknowledging. First, a subset of participants in Experiment 2 was tested in a different setting, and results from Bayesian analyses suggest that the sample size was not large enough to show conclusive results. Second, the level of imaginability of each sentence was not considered. Future studies should explore the impact of each stimulus’ imaginary value and differentiate between high and low imaginability sentences. Besides, it is important for future studies to consider individual mental imagery abilities, since we could expect that differences in imagery abilities might explain whether the instruction to imagine results in a positive or negative effect on memory performance. Third, participants’ confidence level was not measured, a factor that has been shown to modulate the proportion of false memories (Brewer et al., 2005). It might be interesting for future research to assess the confidence levels of both imagination (how accurate they think their generated image was) and retrieval (how confident they are of their response at the final memory test). Finally, the substantial reduction in false recognition errors that we obtained suggests that it may also be worth exploring imaginal encoding as a strategy for the correction of false memories.

Taken together, our findings suggest how an imagery strategy at encoding improves memory. Perhaps the limits of imagining are not the same as the limits of remembering: Our memories are constrained but maybe our imagination is free (McCarroll, 2020). Imaginary encoding surely seems to be a key strategy to create better learning environments, less prone to false memories, and empowering of our veridical memories.

## Data Availability Statement

The datasets presented in this study can be found in the Open Science Framework online repository: https://osf.io/v8apj/?view_only=9969d17536f54053a72be19c050c4767. The names of the repository/repositories and accession number(s) can also be found in the article/Supplementary Material.

## Ethics Statement

The studies involving human participants were reviewed and approved by the Ethics Committee of the Faculty of Psychology of the University of Lisbon. The patients/participants provided their written informed consent to participate in this study.

## Author Contributions

PC conceived the research question and the research design, supervised the data collection, and conducted the preliminary data analysis of Experiment 1. LG-M, MM, AL, and AR developed the research design of Experiment 2. AL programmed and supervised the data collection of Experiment 2. MM conducted the data analysis of Experiments 1 and 2 and wrote the first draft of the manuscript. All authors were involved in the discussion of the theoretical implications of this research. MM, AL, LG-M, and AR revised and approved the final version of the manuscript. PC deceased prior to the development of Experiment 2 and finalization of the paper. All authors contributed to the article and approved the submitted version.

## Conflict of Interest

The authors declare that the research was conducted in the absence of any commercial or financial relationships that could be construed as a potential conflict of interest.

## Publisher’s Note

All claims expressed in this article are solely those of the authors and do not necessarily represent those of their affiliated organizations, or those of the publisher, the editors and the reviewers. Any product that may be evaluated in this article, or claim that may be made by its manufacturer, is not guaranteed or endorsed by the publisher.
