# Supplementary material for: Imagination Reduces False Memories for Everyday Action Sentences: Evidence From Pragmatic Inferences
Source: Front Psychol. 2021 Aug 20;12:668899. doi: 10.3389/fpsyg.2021.668899 (PMC8417559; doi:10.3389/fpsyg.2021.668899)
Supplement: Supplementary file 1 [file Data_Sheet_1.docx]

**Appendix 1. Coding criteria for the 60 pragmatic inference sentences.**

| **Sentence** | **Correct Response** | **Pragmatic Inference** | **Intrusions** |
| --- | --- | --- | --- |
| After dropping off her kids at the school, the mother picked up bread. | picked up | bought | made/cut/gave |
| The charming prince gently put his lips towards Snow White’s cheek. | put his lips towards/approached | kissed | touched/looked/smelled |
| The Sherman tank headed to the flimsy roadblock. | headed to | broke/destroyed/knocked over | went beyond/passed/crossed |
| The snowman vanished when the temperature reached 26ºC. | vanished/disappeared | melted | broke/endured/persisted |
| The flimsy shelf weakened under the weight of the books. | weakened | collapsed/broke down | fell/was loaded/bended |
| The boy lost his balance on the skateboard. | lost his balance | fell of | jumped/bought/took |
| The absent-minded professor didn’t have his car keys. | didn't have | forgot | lost/dropped/left |
| The friendly bartender got extra money from the customer. | extra money/some coins | tips | compliments/news/advice |
| The angry rioter took aim with the stone at the window. | took aim/hit the bull's eye | threw | looked/directed/shouted |
| That radio station liked only hard rock music. | liked | played | listened/talked/had |
| The karate champion hit the cinder block. | hit | broke/smashed/split | kicked/lifted/moved |
| Dennis the Menace sat in Santa’s chair and asked for an elephant. | chair | lap | sleigh/next to/close to |
| The painter knocked over the bucket of black paint. | knocked over | spilled/threw | used/saw/filled |
| The clumsy chemist had acid on his coat. | had | spilled | kept/forgot/avoided |
| The hungry python caught the mouse. | caught | ate | lost/smelled/heard |
| The hungry squirrel was biting the grass. | was biting | was eating | was sitting/was smelling/was playing |
| The safe-cracker put the match to the fuse. | put the match to | lit | found/brought/stole |
| The audiophile adjusted the sound of the stereo system. | adjusted | raised | listened to/checked/stopped |
| The agile cat reached the fish with its claws. | reached | caught | looked/killed/attacked |
| The politician was not very true in his statement about his financial crisis. | not very true | lying | shouting/crying/honest |
| The hill walls weakened under the rainstorm. | weakened | collapsed | made noise/leaked/resisted |
| The rat was attracted by the mousetrap. | attracted | caught | betrayed/dead/lost |
| The birthday boy blew at the candles. | blew at | blew out | put/broke/lit |
| The best student in the class always prepared the next day's subject. | prepared | studied | knew/read/looked |
| Ricardo just wanted to rest for a whole night. | rest | sleep | go out/dance/walk |
| When she looked at her watch, Vera realized she was very late. | looked at her watch | checked the time | was ready/woke up/left home |
| As soon as she reached the beach, Marta spread the towel, put on her sun hat, and lay down on the sand. | sun hat | sunscreen | sunglasses/bikini/headphones/ |
| The policeman saw the car parked in the second row. | saw | emitted a fine to | stopped/found/had |
| Desperate, he approached the bridge. | approached | jumped of | crossed/forgot/looked |
| After a big argument with the boss, João decided to skip work. | skip | quit | complain/change/take a break |
| The night watchman took some coffee from his thermos. | took | drank | dropped/removed/brought |
| King Kong stood on top of the Empire State Building. | stood on top of | climbed | viewed/shouted/hung from |
| The intervention officer knocked on the door. | Knocked on | broke down/destroyed | waited/found/opened |
| The new baby stayed awake all night. | stayed awake | cried | ate/played/slept |
| The noisy guard dog growled at the trespasser. | growled | barked | scared/guided/cried |
| The racecar driver hit the wall in the middle of the race. | hit | crashed into the | passed/stopped/focused |
| After suffering a major injury, the dancer had to take a break from the dance. | take a break | retire | train/compete/show |
| The hypnotist put his fingers together and awakened his client. | put his fingers together | snapped his fingers | raised/touched/closed |
| The thief entered the house. | entered | assaulted | looked/went out/ran to |
| The Christian closes his eyes and remains a few minutes in silence before each meal. | remains a few minutes in silence | prays | thanks/waits/thinks |
| The beautiful blonde walked into the dark room and pressed the switch. | pressed the switch | turned on the light | got scared/got lost/hided herself |
| The scholarly girl leafed through her manual. | leafed through | studied | open/shared/brought |
| The thief entered a store and took a chocolate bar. | took | stole | ate/threw/looked for |
| In the middle of the night the sleepy husband went to get the newspaper and hit the mosquito. | hit | killed | ate/smashed/was bitten by |
| The drunk husband raised his hand to his wife. | raised his hand to | beat up | shouted/threatened/scared |
| The race started when the referee pulled the trigger. | pulled the trigger | fired the gun | beeped/whistled/signed |
| Irritated, the soccer player spoke to the referee. | spoke to | yelled/shouted at | fought/called/listened to |
| After chasing the thief for three blocks, the cop finally reached the thief. | reached | caught | watched/called/stopped |
| The cop shouted at the suspect and fired his gun. | fired his gun | shot him | ran after him/scaped/fined |
| The gymnast made a big mistake and risked the competition. | risked | lost/did not win | abandoned/got injured/sabotaged |
| The kid stung the balloon with a pin. | stung | popped/burst | painted/moved/threw |
| The competition's favorite was the first to pass the race line. | was the first to pass | won | lost/finished/stopped at |
| The ugly sisters of Cinderella asked her to mop the floor. | asked | forced/obliged her to | had to/didn't want to/were about to |
| The captain of the submarine said: -'Submerge submarine!' | said | ordered | afirmed/thought/heard |
| She picked up her favorite pair of shoes and left. | picked up | put on/wore | bought/hide/washed |
| He picked up the phone, seeing that it was his mother who was calling. | picked up | answered | looked to/hung up/forgot |
| It was because she gained 5 kg that she thought about a diet. | thought about | started | stopped/rejected/looked for |
| She stumbled down the stairs. | stumbled | fell | ran/jumped/walked |
| Happy, they analyzed the contract to buy the house. | analyzed | signed | wrote on/celebrated/printed |
| On the day of the interview, she chose her lucky shirt. | chose | wore | washed/forgot/packed |
